# Supplementary material for: Culturally Tailored Community Brain Health Education for Chinese Americans Aged 50 or Above: A Mixed-Methods Open Pilot Study
Source: Geriatrics (Basel). 2025 Apr 14;10(2):58. doi: 10.3390/geriatrics10020058 (PMC12026972; doi:10.3390/geriatrics10020058)
Supplement: Supplementary file 1 [file geriatrics-10-00058-s001.zip › geriatrics-3445680-supplementary.pdf]

Survey Questions Developed or Adapted by the Research Team

Knowledge of Diet (Possible score range: 0 – 7)
Note: The answer in bold represents the correct answer. Each correct response contributes one point to the total knowledge score for the specific domain.

1. Which of the following dietary patterns does not help protect the memory of older adults and prevent dementia?
A. Eating more vegetables and fruits
B. Eating more whole grains
C. Eating more red meat
D. Eating fish

2. If you use a plate of commonly used size for lunch, what proportion of the plate should be filled with vegetables and fruits?
A. Half
B. One-third
C. One-quarter
D. One-fifth

3. What do you think is the best way to supplement calcium?
A. Dark leafy vegetables
B. Grains
C. Bone broth
D. Milk, mini-dried shrimp

4. The finer the rice and flour processing, the more of which vitamin is lost.
A. Vitamin A
B. B vitamins
C. Niacin
D. Vitamin C

5. Vegetables and fruits have similar nutritional components, so if you don't like vegetables, you can replace them with fruits. Do you think this is true or false?
A. True
B. False

6. What is the role of nutrient supplements in supplying nutrients to the body?
A. They can completely replace the diet.
B. Supplements are the main source, and the diet is supplementary.
C. The diet is the main source, and supplements are supplementary.
D. Supplements are not necessary.

7. How often is breakfast recommended?

A. Every day
B. 4-5 times a week
C. 1-4 times a week
D. Never

Knowledge of Exercise (Possible score range: 0 – 7)
Note: The answer in bold represents the correct answer. Each correct response contributes one point to the total knowledge score for the specific domain.

1. Which of the following is not an aerobic exercise?
A. Jogging
B. Brisk walking
C. Cycling
D. Dumbbell lateral raise

2. According to the latest exercise guidelines, what is the minimum recommended time for moderate-intensity aerobic exercise per week?
A. 150 minutes
B. 120 minutes
C. 100 minutes
D. 180 minutes

3. Moderate-intensity aerobic exercise means that you exercise hard enough to raise your heart rate to the corresponding range for your age and start sweating. Do you think this statement is correct?
A. Yes
B. No

4. Which of the following is a high-intensity aerobic exercise?
A. Yoga
B. Brisk walking
C. Singles tennis
D. Washing dishes

5. According to the latest exercise guidelines, which of the following is correct about recommended resistance training?
A. Resistance training should be performed for at least 2 days per week.
B. Resistance training should be performed for at least 3 days per week.
C. Resistance training should be performed for at least 4 days per week.
D. Resistance training should be performed for at least 5 days per week.

6. According to the latest exercise guidelines for middle-aged and older adults, how many times a week is recommended for balance improvement activities?

A. 1 time
B. 2 times
C. 3 times
D. 4 times

7. Which of the following is not an activity to improve balance?
A. Walking backward
B. Single-leg standing
C. Using a wobble board
D. Jumping rope

Knowledge of Common Health Check (Possible score range: 0 – 8)
Note: The answer in bold represents the correct answer. Each correct response contributes one point to the total knowledge score for the specific domain.

1. How often do you think it is reasonable to have a routine physical examination in a year?
A. Once
B. Twice
C. Three times
D. Four times

2. If you go to the hospital for a check-up and find that your blood pressure is higher than the normal range after one measurement, can you determine that you have hypertension?
A. Yes
B. No

3. Which of the following indicators suggests an increased risk of cardiovascular disease?
A. High-density lipoprotein cholesterol (HDL)
B. Low-density lipoprotein cholesterol (LDL)

4. Which of the following indicators decreasing suggests a risk of iron-deficiency anemia?
A. Hemoglobin (Hb) B. Platelets (PLT) C. White blood cells (WBC) D. Blood lipids (TG)

5. Which of the following is not an indicator of liver function?
A. Alanine transaminase (ALT)
B. Platelets (PLT)
C. Aspartate transaminase (AST)
D. Alkaline phosphatase (ALP)

6. Which of the following is an indicator of kidney function?
A. Alanine transaminase (ALT)
B. Platelets (PLT)
C. Creatinine (Cr)

D. Blood lipids (TG)

7. If serum uric acid (UA) is higher than the normal range, what disease may be the possible cause?
A. Nephritis
B. Urinary tract infection
C. Hepatitis
D. Gout

8. If serum creatine kinase (CK) is higher than the normal range, what disease may be the possible cause?
A. Myocardial infarction
B. Diabetes
C. Acute hepatitis
D. Alzheimer's disease

Knowledge of Brain health (Possible score range: 0 – 8)
Note: The response options include True or False. The answer in parenthesis represents the correct answer. Each correct response contributes one point to the total knowledge score for the specific domain.

1.Brain health affects cognition, memory, thinking, problem solving, orientation, and speech. (True)

2.Laughing and humor are ways to manage stress. (True)

3.Sleep quality has no influence on brain health. (False)

4.A more physical lifestyle might slow cognitive decline. (True)

5.The body's internal clock shifts as people age. (True)

6.Smoking and drinking in excess are not good to your brain health. (True)

7.A plant-based diet that is rich in a variety of fruits and vegetables, particularly green-leafy vegetables, and berries, is associated with better brain health. (True)

8.Organize a regular virtual game night or book club is one of the ways to be social and maintain brain health. (True)

9.Diabetes damages blood vessels throughout your body, including your brain. It also increases risk of heart disease, memory problems and Alzheimer's disease. (True)

10.Avoiding multitasking could help people improve memory. (True)

Behavioral motivation of lifestyle changes for dementia risk prevention (Possible score range: 5-35)

Note: Responses were based on the level of agreement with the following statement, which include 1-strongly disagree, 2-disagree, 3-neither agree nor disagree, 4-agree, and 5-strongly agree. First, responses for the first four items were reverse coded. Second, after reverse coding, the sum of the responses was used to measure behavioral motivation, with a higher score indicating a higher level of behavioral motivation.

1. I am too busy to change my lifestyle and health habits

2. My financial situation does not allow me to change my lifestyle and behavior

3. Family responsibilities make it hard for me to change my life and behavior

4. Changing lifestyle and behavior interfere with my schedule

5. I am paying attention to my lifestyle and behavior to reduce dementia risk

6. I often think about my brain health

7. I am confident that my lifestyle and behavior can reduce the risk of developing dementia.
